# Axial Connectivity of the Lung Microbiome: A Review of Interorgan Crosstalk

**DOI:** 10.1002/cph4.70269

**Published:** 2026-09-18

**Authors:** Mathangi Palanivel, Jayanth Kumar Narayana, Sanjay H. Chotirmall

**Affiliations:** ^1^ Lee Kong Chian School of Medicine Nanyang Technological University Singapore Singapore; ^2^ Department of Respiratory and Critical Care Medicine Tan Tock Seng Hospital Singapore Singapore

## Abstract

The classic organ‐centric model of human physiology is rapidly giving way to a unified approach embracing the human body as an integrated network of bidirectional interorgan communication. The human microbiome serves as a fundamental mediator of this shift, regulating immune homeostasis, barrier integrity and metabolic signaling across organs. While the lung maintains a characteristic low‐biomass microbiome in dynamic equilibrium, any disruption primes local and systemic immune responses contributing to disease. Beyond the well‐described gut‐lung axis, crosstalk between the lung and other distal organ systems is lesser recognized; however, increasingly acknowledged as a key under‐appreciated contributor to respiratory disease including extrapulmonary complications. This review synthesizes current established evidence on axial connectivity of the lung microbiome across the gut, brain, skin, heart, kidney, and liver, and finds that such crosstalk is predominantly, though not exclusively gut‐mediated. Direct lung‐organ interactions are described for several axes; however, they remain preliminary. Across these lung‐axial systems, common pathophysiological mechanisms emerge, including dysbiosis‐induced depletion of microbial metabolites, immunomodulation, and barrier perturbations, all linking pulmonary disease with neuroinflammation, gastrointestinal deficits, and cardiac, renal, hepatic, and dermatological abnormalities. We assess how therapeutic modulation of interorgan systems offers promising avenues for improved risk stratification and novel therapeutics. Recognizing microbiome‐mediated pulmonary‐organ crosstalk represents an emerging conceptual framework in respiratory medicine, repositioning microbial communities as key modulators of extrapulmonary disease.

## Graphical abstract

The human microbiome mediates immune homeostasis and metabolic signaling across organs. Beyond the gut‐lung axis, crosstalk between the lung and other organ systems is less recognized. This review assesses the axial connectivity of the lung microbiome across the gut, brain, skin, heart, kidney, and liver.

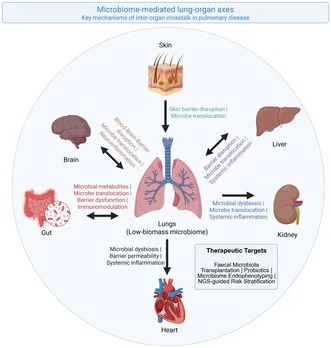

## 1 Introduction

The classic model of human physiology is rapidly evolving from a single organ‐centric approach toward a holistic, unified perspective of interacting organs and tissues (Komaru et al. 2024; Priest and Tontonoz 2019; Huh and Veiga‐Fernandes 2020). Recent studies reveal that organs are not discrete entities and are involved in constant, bidirectional communication commonly referred to as “axes” (Komaru et al. 2024; Dora et al. 2024; Bajinka et al. 2022; Herrero et al. 2020; Rocha et al. 2024; Deng et al. 2025). Specifically, an acknowledgement of the human microbiome, encompassing the collection of microorganisms and their genetic material within the human body, serves as a fundamental influence on health and disease and is pivotal to this paradigm shift (Budden et al. 2017). The human microbiome has critical roles in maintaining homeostasis and providing multifarious benefits to the host, including immune regulation and nutrient absorption (Moens and Veldhoen 2012). Despite the emerging significance of microbiomes in preserving health, their disruption may drive disease; for example, pulmonary microbial dysbiosis contributes to lung disease through mechanisms including impaired epithelial barrier integrity, dysregulated local immune responses, and modulation in inflammatory signaling (Althani et al. 2016; Young 2017; Li, Li, and Zhou 2024), while dysfunction in organs distal to the lung, such as the gut, through propagation of microbial metabolic and immune signals, has been found to contribute to such pathogenesis (Ahlawat et al. 2021; Levy et al. 2017; Sencio et al. 2021). Such crosstalk is now implicated across the spectrum of human disease, including pulmonary and neurodegenerative diseases, metabolic syndrome, and overall systemic health (Priest and Tontonoz 2019; Carata et al. 2025; Li et al. 2025). Consequently, the microbiome represents a promising target for therapeutic modification, with most notable advances including fecal microbiota transplantation (FMT) and microbial endophenotyping through next‐generation sequencing contributing to improved risk stratification, prognostication, and disease outcomes (Chotirmall et al. 2022; Li, He, et al. 2021).

The lung is characterized by a large surface area continually exposed to the external environment acting as a hub for interorgan networking (Budden et al. 2017). Historically, the lungs were incorrectly regarded as sterile (Wypych et al. 2019; Dickson et al. 2016). However, it is now appreciated that the lungs accommodate a distinctive, low‐biomass microbiome maintained at a dynamic equilibrium determined by microbial immigration through inhalation, micro‐aspiration, and microbial eradication through mechanisms including cough, mucociliary clearance, and regional growth conditions within the lung (Dickson and Huffnagle 2015; Dickson et al. 2014). Disruption of the pulmonary microbiome is recognized to play key roles in several respiratory diseases by coordinating local and systemic immune responses and enabling host defenses to maintain homeostasis. Additionally, interactions between pulmonary and gut microbiomes have been shown as crucial in mediating such responses (Li, Li, and Zhou 2024).

**FIGURE 1:**
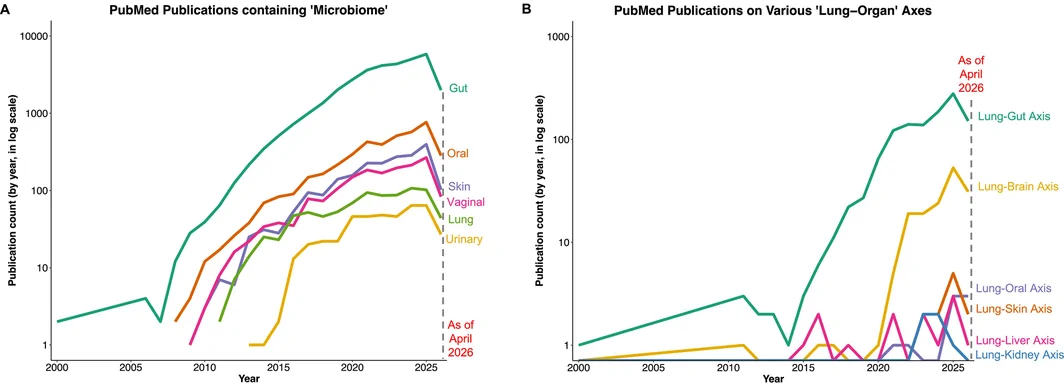
(A) Count of PubMed publications (by year, in log scale) containing the keyword “microbiome” over time. The six individual organ microbiomes, with the keywords, “gut microbiome”, “oral microbiome”, “skin microbiome”, “vaginal microbiome”, “lung microbiome”, and “urinary microbiome” were used to retrieve the publications. (B) Count of PubMed publications (by year, in log scale) addressing “lung‐organ” axes over time. The following lung‐organ axis term pairs were used to retrieve publications: “lung‐gut axis” OR “gut‐lung axis”, “lung‐brain axis” OR “brain‐lung axis”, “lung‐oral axis” OR “oral‐lung axis”, “lung‐skin axis” OR “skin‐lung axis”, “lung‐liver axis” OR “liver‐lung axis”, “lung‐kidney axis” OR “kidney‐lung axis”. Gray dotted lines in A and B represent the final datapoint for each plot which depicts publications retrieved as of April 2026. The searches were performed on PubMed using the search terms listed above as free‐text queries entered directly into the PubMed search bar, which by default searches across title, abstract, MeSH terms, and other indexed fields. The searches include all available years up to the retrieval date of 23 April 2026. The records were imported into EndNote, and duplicate records were identified and removed using EndNote's in‐built “Find Duplicates” function prior to tabulation of publication counts per year.

This “gut‐lung” axis enables bidirectional communication, permitting gut microbiota to impact respiratory health and vice‐versa (Dang and Marsland 2019; Özçam and Lynch 2024; Wang et al. 2023; Narayana et al. 2023). Although this axis is now well established, there is a necessity to investigate other relevant “lung‐organ” axes to better appreciate how remote influences may affect the lungs and lung disease (and vice versa) through common mechanisms. Assessing the rate of this emerging interest provides a useful benchmark for how the field's focus has shifted from studying individual organ microbiomes toward investigating connections between them, and for recognizing where synthesis of existing evidence is required. We therefore examined PubMed publication trends over time across six key individual organ‐based microbiomes (Figure 1A), where a steady increase in studies over time indicates growing interest in the field of microbiome research. Figure 1B illustrates PubMed publications (by year) retrieved over time focused on pulmonary axes—microbiome publications across other organ systems, which emerge as a smaller, and more recent subset, underscoring the relevance of consolidating this literature. Together, these trends provide support for the premise of this review, that research on cross‐organ microbiomes remain in the early stage relative to the study of discrete organ microbiomes, hence highlighting the need to synthesize this evidence.

This review seeks to integrate our current understanding of microbial axes linking the lung to other organ systems within the framework of common pulmonary diseases, focusing on mechanisms of interaction mediated by the microbiome and microbial products. Across these axes, the gut consistently emerges as a key intermediary through which the lung communicates with distal organs, while direct, non‐gut‐mediated lung‐organ signaling is also described, albeit evidence remaining relatively preliminary and less extensively characterized. We will outline modes of pulmonary axis crosstalk, with particular focus on the established gut‐lung and lung‐brain axes, before exploring emerging evidence linking the lung, via the gut, with the skin, kidney, liver, and heart. Beyond effects on pulmonary diseases itself, disruption of these microbial axes may also contribute to extrapulmonary disease manifestations in linked organs, a relationship that is highlighted in this review. We will discuss future perspectives, highlighting therapeutic opportunities relating to targeting microbiomes to address common diseases.

## 2 The Emerging Recognition of Pulmonary‐Axial Crosstalk in Respiratory Disease

**FIGURE 2:**
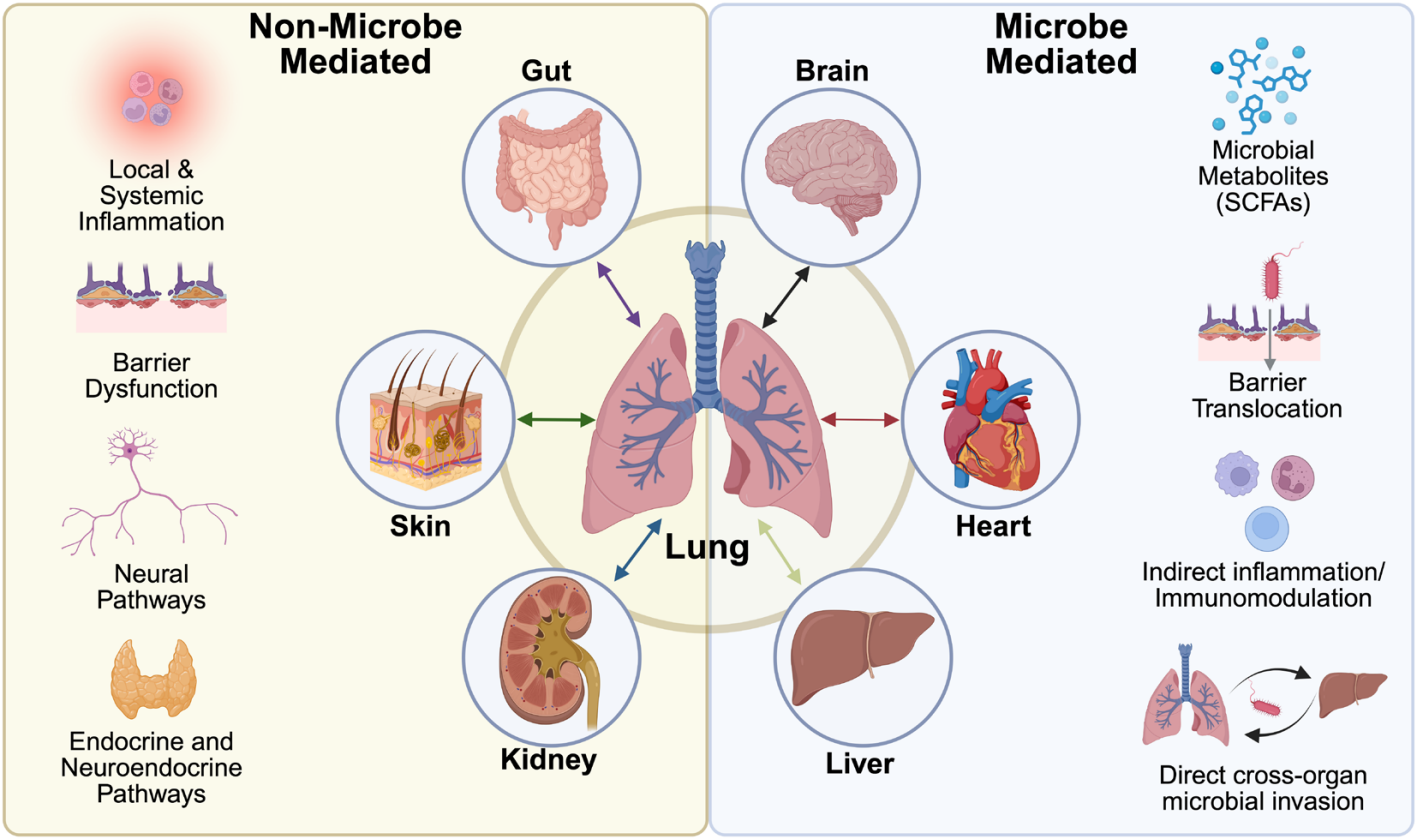
A summary of the modes of pulmonary‐axial crosstalk. Crosstalk between the lungs and major organs can occur through inflammatory processes, barrier dysfunction, neural pathways, endocrine, and/or neuroendocrine pathways independent of microbes or processes arising from microbial involvement including direct cross‐organ invasion and barrier traversal, transmittance of metabolites secreted by microbes or inflammation, and immunomodulation triggered by microbes and their products. Figure created in https://BioRender.com.

Cross‐organ communication across lung‐organ axes is regulated through various mechanisms, termed “inter‐organ crosstalk”, encompassing an exchange of signals including immune cells, cytokines, neural inputs, and perturbations to barrier function (Li et al. 2025). Signal exchange between the lungs and other organs may occur through microbe‐mediated or non‐microbial routes (Liu et al. 2026; Chakraborty et al. 2024; Gillan et al. 2025) and Figure 2 illustrates the varying modes of lung‐organ crosstalk described to date. Here, we outline several major mechanisms of such interplay between the lungs and other vital organs including the gut, brain, skin, kidney, liver, and heart.

### 2.1 Non‐Microbial Mechanisms of Pulmonary‐Axial Crosstalk

#### 2.1.1 Local and Systemic Inflammation

Inflammatory mediators including IL‐6, IL‐1β, TNF‐⍺, CRP, and HMGB1 are essential to facilitate immune responses between the lungs and other organ systems (Li et al. 2025). The lungs exhibit crosstalk with several organs including the gut, brain, skin, kidneys, liver, and heart through local and systemic inflammatory processes which can affect distant organs.

Much of this crosstalk is gut mediated. For instance, systemic proinflammatory mediators in chronic obstructive pulmonary disease (COPD) promote cross‐organ inflammation across the lung, gut, and skin, with significant correlation observed between biophysical characteristics of the skin and serum inflammatory markers suggestive of compromised skin health in COPD (Majewski et al. 2017). Immune imbalance through cytokine upregulation in the brain is implicated in instances of acute lung injury (ALI)‐linked neurological damage through this gut‐mediated route (Ziaka and Exadaktylos 2024a).

Direct, non‐gut‐mediated inflammatory crosstalk is also evident between the lungs and discrete organs. Systemic IL‐6 and lung TGF‐β may initiate Th‐17‐polarized inflammatory responses in the gut of individuals with COPD (Wang et al. 2023). Asthma is associated with an elevated risk of dementia and Alzheimer's Disease, though the underlying mechanisms remain poorly defined in comparison to the gut‐mediated ALI pathway (Joh et al. 2023). In parallel, damage to the liver, kidneys, and/or skin can cause local inflammation in the respective organ, prior to aggravating pulmonary inflammation in a systemic manner (Herrero et al. 2020; Deckers et al. 2018; Li et al. 2022). Dysfunction to the brain, such as during instances of stroke or traumatic brain injury (TBI), may perpetuate systemic inflammatory responses affecting the lungs. In TBI‐related ALI, systemic hypoxemia can aggravate the brain's metabolic crisis, impairing brain autoregulation due to altered lung hemodynamics and resulting in a secondary neuronal insult from proinflammatory cytokine trafficking (Mascia 2009).

#### 2.1.2 Barrier Dysfunction

Damage to epithelial or endothelial barriers facilitates the systemic spread of mediators and pathogens. Interactions between the lungs and other organs are frequently noted to be sustained through such compromised and/or dysfunctional barriers (Herrero et al. 2020; Rocha et al. 2024; Deng et al. 2025; Dickson et al. 2016; Domenech et al. 2017; Greene et al. 2024). A key instance is skin barrier dysfunction in dermatitis which triggers T‐cell and Th‐17 cell‐dependent immune responses leading to skin and lung inflammation (Saunders et al. 2020). Protein mediator release by injured kidneys or enhanced blood endotoxin levels in individuals with liver disease can induce prospective enhancement to endothelial barrier permeability promoting pulmonary oedema and respiratory failure (Siore et al. 2005; Khamissi et al. 2022). In the brain, lipopolysaccharide (LPS)‐induced acute respiratory distress syndrome (ARDS) disrupts the blood–brain‐barrier (BBB) through downregulation of endothelial tight junction protein expression resulting in reduced cerebral perfusion (Hernandez et al. 2023). Similarly, ARDS raises alveolar‐capillary barrier permeability that leads to a hypoxic‐induced pulmonary vasoconstriction and right ventricular ischemia (Rocha et al. 2024). In cigarette smokers with COPD, epithelial barrier dysfunction, ensuing from an increased airway epithelial permeability may facilitate bacterial translocation into the gastrointestinal (GI) tract and induce inflammation.

#### 2.1.3 Neural Pathways

The lung additionally may communicate with distant organs through specialized circuits transmitting sensory, autonomic, and inflammatory signals. This is evident in lung‐brain, lung‐gut‐kidney, and lung‐gut signaling (Li et al. 2025). Direct neural crosstalk is evident between the lung and the brain, and within the lung‐gut axis itself. Lung‐brain signaling occurs via vagus afferents (Huang et al. 2025), whereby corresponding pulmonary sensory neurons trigger serotonin release from the dorsal raphe nucleus, suggesting potential lung‐vagus nerve‐brain communications through axes cited in major depressive disorder (Huang et al. 2023). Vagal afferents from the GI and respiratory systems converge across the nucleus of the solitary tract rather than strictly remaining compartmentalized, offering a neuroanatomical substrate for direct lung‐gut signaling at the brainstem level (Bassi et al. 2022). Interactions between the lung and kidney, at the neural level, are not direct; however, the renal afferent vagus nerve possesses a capability to detect pulmonary injury and participate in a neuroimmune anti‐inflammatory reflex affecting kidney function and thus contributing to communication between the lungs and kidneys.

#### 2.1.4 Endocrine and Neuroendocrine Signaling

Beyond neural circuitry, the lung itself acts as an active endocrine organ, expressing hormone receptors and secreting mediators with both local and systemic effects (Iglesias 2025; Abelson et al. 2010). Pulmonary neuroendocrine cells display this dual function, as they are the sole lung epithelial cells that are directly innervated by sensory neurons, which can detect airway stimuli and release neuropeptides such as serotonin and calcitonin gene‐related peptide (CGRP), providing sensory communication through which local airway signals could affect distant neural and endocrine outputs (Thakur et al. 2024). Additional lung‐derived mediators, such as leptin and endothelin‐1, further diversify this endocrine role by contributing to functions such as vascular tone regulation and immune modulation in other organ systems (Iglesias 2025; Davie et al. 2002; Huertas et al. 2012).

This neuroendocrine signaling is closely associated with the hypothalamic‐pituitary‐adrenal (HPA) axis, through which neuroregulatory molecules, released following lung injury, may activate the axis and influence the central nervous system (Abelson et al. 2010). Reciprocally, glucocorticoids derived from the HPA axis could exert an immunoregulatory effect on the lung, and the dysregulation of this circuit has been proposed to be a contributor to the systemic and neurocognitive complications arising in chronic respiratory diseases such as COPD (Yu et al. 2025).

Organ‐specific endocrine pathways further connect the lungs to the kidneys and heart. The lung is the key site of angiotensin‐converting enzyme (ACE) activity within the renin‐angiotensin system, converting circulating angiotensin I to II (Orfanos et al. 1999). Together with the counterregulatory ACE2/angiotensin‐(1‐7)/Mas receptor axis, this pulmonary renin‐angiotensin activity influences vascular tone, inflammation, and lung fibrosis (Lang et al. 2025), and separately through the systemic circulation and in the kidneys where this circulating angiotensin II is absorbed along parallel local renin‐angiotensin components (Yang and Xu 2017).

### 2.2 Microbial‐Mediated Mechanisms of Pulmonary‐Axial Crosstalk

Microbial mediation is increasingly recognized as a significant, although not exclusive, contributor to the complex crosstalk between the lungs and other organ systems. The microbiome, microbes within, and their products including LPS may translocate across compromised barriers influencing distal organs such as the brain and their resident immune cells including microglia to promote neuroinflammation (Li et al. 2025; Huang et al. 2025). Microbial metabolites, particularly short chain fatty acids (SCFAs), largely produced by gut bacteria, circulate systemically modulating immune cell function and bone marrow hematopoiesis while shaping pulmonary immune responses (Dang and Marsland 2019; Li, Yao, et al. 2024). Pathways including CARD9‐Th17 signaling, triggered by microbial components, remain critical to orchestrating immune cell migration and inflammatory modulation in distal tissues (Drummond and Lionakis 2016). In addition, pathogen‐associated molecular patterns (PAMPs) are a key mechanism driving interorgan communication that are recognized by pattern recognition receptors (PRRs) on host cells, triggering immune consequence (Yang, Mao, et al. 2025). Such interactions, irrespective of their initiation from resident lung microbiota or translocated gut bacteria, accelerate innate immune cascades releasing pro‐inflammatory mediators which propagate, systemically impacting organs including the brain and kidneys, contributing to neuroinflammation and endothelial dysfunction respectively. Furthermore, microbial dysbiosis results in promoting barrier dysfunction, increasing intestinal permeability, for instance, facilitating translocation of microbes and their associated metabolites (Li et al. 2025; Huang et al. 2025). Such microbial‐derived signals may amplify non‐microbial interplay indirectly through enhancing systemic inflammation and neural inter‐organ communication, creating a loop connecting pulmonary health to broader multi‐organ pathology.

**FIGURE 3:**
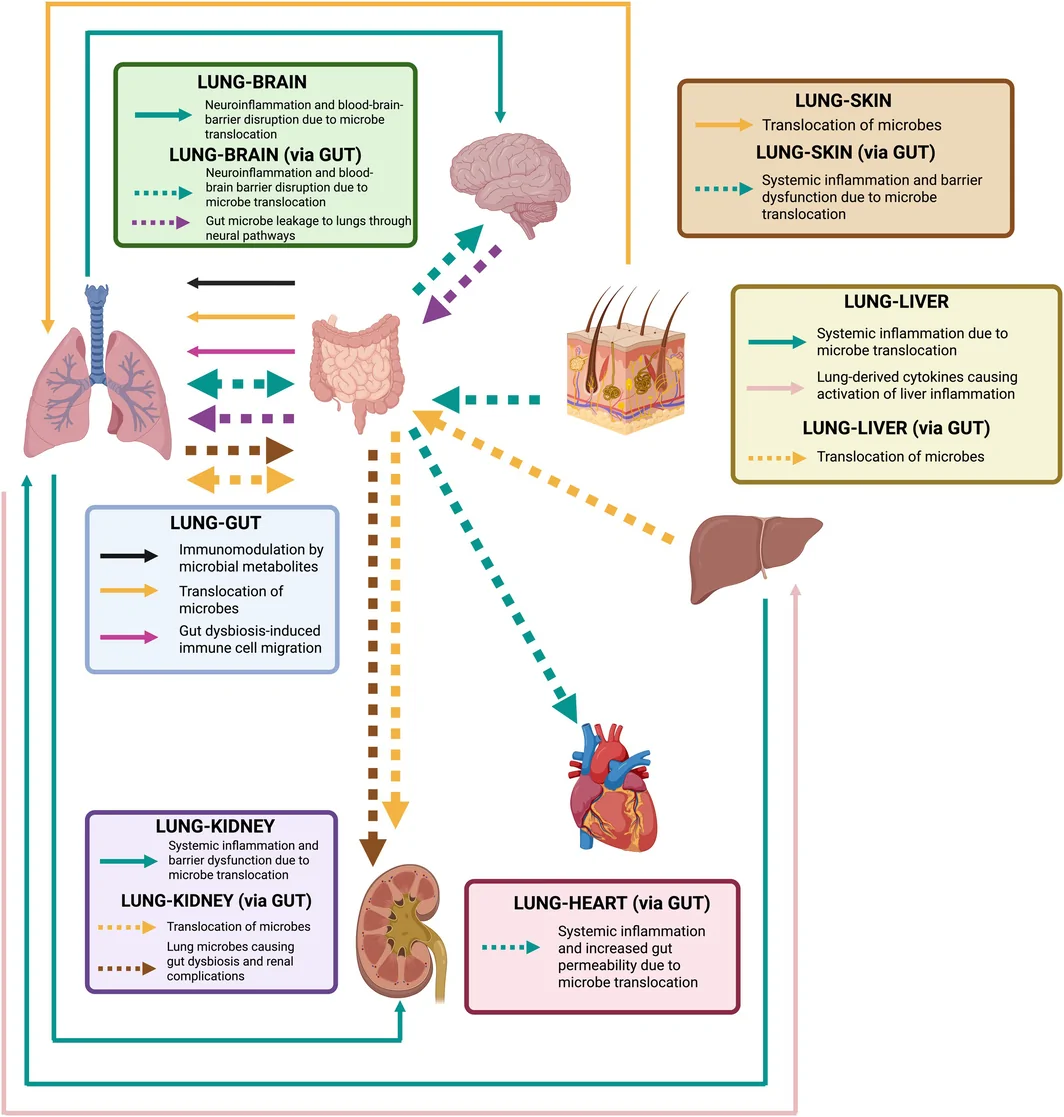
Schematic depicting lung‐organ crosstalk observed between the lung and gut, brain, heart, skin, kidneys, and liver, respectively. All systemic organs communicate with the lung through the gut, with only the gut, brain, skin, liver, and kidney establishing direct crosstalk with the respiratory system. Several links are observed between the lung and other organ systems whereby thin, solid and thick, dotted lines represent direct and indirect microbe‐mediated pathways through which such crosstalk occurs, respectively. Yellow lines (both solid and dotted) represent the translocation of microbes due to an increased barrier permeability of either the gut or skin. Brown dotted lines depict gut microbial and metabolite disruption following infection, leading to renal complications. Purple dotted lines signal gut microbial leakage to the lungs initiated due to a degeneration of brain nociceptors innervating the gut, thereby compromising gut barrier integrity. Figure created in https://BioRender.com.

Lung‐organ crosstalk in vivo may be substantially mediated by microbe‐associated mechanisms, whereby microbial products and metabolites circulate systemically and interact directly with distant tissues and organs (Budden et al. 2017; Li, Li, and Zhou 2024; Zhu et al. 2018). These mechanisms, encompassing direct translocation of microorganisms through the bloodstream or lymphatic system, circulating microbial metabolites and products such as short chain fatty acids (SCFAs), and correlated changes in microbiota composition across distant epithelial barriers, are collectively referred to as “microbial traffic”, which simultaneously promotes inflammation, perturbs barrier integrity, and modulates neuro‐immune signaling (Trompette et al. 2014; Braniste et al. 2014; Erny et al. 2015). These microbe‐mediated effects, both direct and indirect, extend to additional processes inclusive of metabolic dysfunction, immune cell migration, and altered metabolic signaling across all organ systems. A summary of the key axes between the lungs and other key organs is summarized in Figure 3, with focus on microbe‐mediated mechanisms of inter‐organ communication. Each of the respective axes, including between the lung and other organs systems including the gut, brain, skin, kidney, liver, and heart, are explored, based on current evidence, in relation to common respiratory diseases.

## 3 Pulmonary‐Organ Crosstalk: An Under‐Recognized Contributor to Lung Disease?

### 3.1 Established Axes: The Gut‐Lung and Lung‐Brain Relationship

#### 3.1.1 Gut‐Lung Axis

The gut‐lung axis refers to the bidirectional communication between gastrointestinal microbiota and the respiratory system which may affect health and disease, with gut microbial disruption increasingly associated with the development and progression of lung diseases, including asthma, COPD, pulmonary fibrosis, bronchiectasis, and/or cystic fibrosis, as well as respiratory infections such as COVID‐19, pneumonia, and tuberculosis (Narayana et al. 2023; Ramar et al. 2025; Bowerman et al. 2020; Li, Wu, et al. 2024; Chen et al. 2022; Bernard‐Raichon et al. 2022; Salameh et al. 2023; Barrack et al. 2024; Zhang, Luan, et al. 2025; Ngo et al. 2024; Ding et al. 2022).

Microbes and their products mediate communication between the gut and lung. Specifically, gut microbes generate immunomodulatory metabolites such as short‐chain fatty acids (SCFAs) that influence T‐cell differentiation, regulate cytokine production and enhance mucosal barrier integrity in both organs (Kim et al. 2014; Mao et al. 2022; Otake et al. 2025). By promoting immune tolerance and mitigating detrimental inflammation, SCFAs have crucial protective roles; however, gut dysbiosis drives reductions in SCFAs, thereby exacerbating inflammation and weakening host defenses (Zheng et al. 2022). For instance, in COPD, reduced gut microbial diversity and SCFA levels are associated with increased lung inflammation, accelerated airflow impairment and airway remodeling (Otake et al. 2025; Li, Dai, et al. 2021). In pulmonary hypertension, dysbiosis in SCFA‐producing gut bacteria, in both humans and animal models, associates with modified pulmonary vasculature (Zhang, Liang, et al. 2025; Callejo et al. 2018; Moutsoglou et al. 2023). Acetate‐producing bacteria were reduced in pulmonary hypertensive rats and it is speculated that the resultant increase in sympathetic activity with this disease may compromise gut barrier function, consequently altering gut microbiota (Callejo et al. 2018). Comparably, in patients with pulmonary hypertension, a reduction is observed in the relative abundance of several bacterial taxa expressing genetic codes linked to SCFA generation. Interestingly, in this setting, gut dysbiosis is posited as a causative factor for pulmonary hypertension, while a reduction in SCFAs may provide an optimal environment for pulmonary arterial smooth muscle cell propagation and fibrosis. Moreover, increased gut permeability may allow for the translocation of harmful endotoxins and/or gut bacteria to the lungs, permitting a heightened inflammatory state in the vasculature (Moutsoglou et al. 2023).

Furthermore, unlike microbial metabolites (such as SCFAs), microbes themselves can directly facilitate communication between the gut and lungs, influencing epithelial barrier dysfunction and pulmonary inflammation, potentially through the impact of commensals (Sagar et al. 2014). It is noteworthy that the pili‐like protein, a highly enriched outer membrane component of *Akkermansia*, is identified as beneficial to gut barrier function and immunological homeostasis (Ottman et al. 2017). Increased barrier permeability permits bacterial components such as LPS and whole bacteria to translocate from the gut into the systemic circulation, inducing low‐grade systemic inflammation which directly impacts the lungs, observed in cases of acute lung injury (ALI) (Tang et al. 2021; Zhang et al. 2019; Tan et al. 2020). Additionally, gut dysbiosis including reductions in *Bifidobacterium* and *Faecalibacterium*, associates with increased intestinal permeability (Leclercq et al. 2014; Al‐Sadi et al. 2021). In asthma, loss of these gut taxa including *Bifodobacterium*, *Faecalibacterium*, and *Akkermansia* appear to associate with greater disease severity disrupting immune regulation and heightening allergic sensitization possibly through disrupted epithelial barrier of the intestine (Hevia et al. 2016; Fukui 2016; Demirci et al. 2019). Importantly, similar observations in COVID‐19 infection have revealed gut‐lung axis disruption associated with disease severity, the reduced presence of beneficial gut anaerobes, heightened gut permeability, microbial translocation, and unique microbe‐metabolite‐cytokine networks which influence the immune response and promote extrapulmonary complications (Bernard‐Raichon et al. 2022; Nagata et al. 2023).

Our immune system establishes a clear communication channel between the gut and lungs. Fundamentally, the concept of common mucosal surface between the two organ systems is posed to be part of a unified immune network, coordinating immune responses across distant locations (Dora et al. 2024; Du et al. 2023). Immune cells, including T‐cells, dendritic cells, and macrophages, are activated in the lymphoid tissue of the gut in response to gut dysbiosis, following which these cells migrate through the bloodstream and lymphatic system to the lungs, which in turn influences the local immune environment and its response to inhaled pathogens and/or allergens (Song et al. 2024). For instance, gut dysbiosis alters the differentiation of critical immune cells such as T‐regulatory (Tregs) and T‐helper 17 (Th17) cells, both crucial in mediating inflammation across various lung conditions including ARDS and COPD (Song et al. 2024; Ziaka and Exadaktylos 2024b).

Finally, while gut microbiomes serve as potential causative factors across a variety of pulmonary diseases, this is not always in one direction. Rather, a bidirectional link between these organ systems is notable, establishing a feedback loop where dysfunction in one may promote, sustain and/or exacerbate pathology in the other (Wang et al. 2023). For example, pulmonary fibrosis drives gut microbial alteration, including an increased *Lachnospiraceae* and altered tryptophan metabolism, while reciprocally, transplantation of these fibrotic‐related microbiota into the gut of pseudo germ‐free mice via oral administration worsens lung pathology confirming the importance of bidirectional microbiome signaling (Li, Wu, et al. 2024; Gong et al. 2021; Gurczynski et al. 2023). In bronchiectasis, dysregulated gut‐lung microbial networks (bidirectional) in high‐risk individuals are characterized by an increased lung *Pseudomonas* and gut *Bacteroides*, which associate with poorer clinical outcomes. Animal experiments reveal that respiratory infection such as *Pseudomonas aeruginosa* may lead to gut dysbiosis and conversely alterations in gut microbiota, which impact the lung, a conceptual framework not limited to bronchiectasis (Narayana et al. 2023; Wang, Shen, et al. 2025). Emerging research in animal models of pneumonia or ARDS reveals that pulmonary infections (e.g., *Influenza* or *Klebsiella*) can trigger gut microbial and/or metabolic shifts, while gut dysbiosis aggravates lung infection outcomes. Moreover, translocation of microbes between the gut and lungs and vice versa is observed in sepsis and ventilator‐associated pneumonia, which in turn contributes to systemic inflammation, further exemplifying the bidirectional relationship between these two organ systems (Wolff et al. 2021; Zheng et al. 2023; Gao et al. 2025). Gut‐to‐lung translocation of *Escherichia coli* and *Burkholderia cenocepacia* is observed in patients with ventilator‐associated pneumonia, potentially inducing additive inflammation through membrane and surface polysaccharide components, alongside known mechanisms such as that through pathogen‐associated molecular patterns (Gao et al. 2025; Drevinek and Mahenthiralingam 2010).

Taken together, the gut‐lung axis represents a significant factor in the development and progression across a number of lung diseases, highlighting the gut microbiome as a potential source of biomarkers and an attractive target for novel therapeutic strategies targeted at the lung.

#### 3.1.2 Lung‐Brain Axis

Seminal studies uncover a microbiome‐mediated lung‐brain axis, suggesting that microbial populations in the airways may affect brain inflammation and functionality (Bajinka et al. 2022). Direct connections between the lung and brain are evident; for instance, modulation of lung microbiomes has direct effects on pain alleviation in mouse models of migraine, likely through neural pathways. To further interrogate these observations, Liu et al. administered neomycin intratracheally to a migraine‐induced mouse model to alter the lung microbiome. This activated the pulmonary vagus nerve through brain‐derived neurotrophic factor‐tropomyosin receptor kinase B pathways in the lung, stimulating serotoninergic neurons in the dorsal raphe nucleus, and consequently increasing serotonin levels and relieving pain (Liu et al. 2025). Moreover, the direct translocation of microbes from the lungs to the brain in the event of bacterial infections is reported (Bajinka et al. 2022; Ma et al. 2023; Villalba et al. 2023). Importantly, in a mouse model of severe pneumonia, intratracheal delivery of LPS significantly elevated levels of inflammatory cytokines in the lung alongside corresponding observations including disrupted lung‐blood and blood–brain barriers (BBB), the presence of endogenous lung bacteria in the brain, and an overall interference of brain homeostasis (Ma et al. 2023). A similar study explored immune‐mediated signaling between the lung and brain through *Pseudomonas aeruginosa* inoculation of mice, where lung infection resulted in alveolar‐capillary barrier injury and cytokine‐induced BBB leakage and consequential neuroinflammation (Villalba et al. 2023).

Importantly, however, and frequently recognized, is that the connection between lung and brain is largely facilitated indirectly by involvement of the gut. For instance, in ARDS, often characterized by gut dysbiosis and increased gut gram‐negative bacteria, it is shown that FMT from ARDS patients into antibiotic‐treated recipient mice results in elevated systemic LPS, blood–brain‐barrier disruption, increased microglial activation, astrocyte proliferation, and cognitive impairment, establishing a mechanistic link between lung disease‐associated gut dysbiosis and neuroinflammation through the gut‐brain axis (Zheng et al. 2023). Similarly, gut dysbiosis, established following COVID‐19, is recognized as a risk factor for neurodegeneration (Dolatshahi et al. 2021). The infection of ACE2‐rich intestinal enterocytes by SARS‐CoV‐2 disrupts dopamine‐related metabolic pathways, offering a potential mechanistic explanation for long‐COVID brain symptoms, involving neuropsychiatric symptoms that continue, recur, or evolve several weeks or months after an initial COVID‐19 infection (Nataf and Pays 2021). Following brain injury, stroke‐associated pneumonia risks correlate with reductions noted in gut microbes which produce SCFAs alongside an enrichment of pathogenic taxa, which when considered improve predictive models and illustrate gut‐lung‐brain axial connectivity (Li et al. 2023). In severe traumatic brain injury (TBI), nociceptor‐mediated translocation of gut microbiota to the lung drives pulmonary infections; while the restoration of TRPV1 signaling through capsaicin administration reduces lung bacterial load accentuating the neural‐microbial pathways linking the brain and lungs (You et al. 2024). Moreover, mechanically ventilated TBI patients with dominant lung *Staphylococcus* and *Acinetobacter*, upon hospital admission, have an increased risk of ventilator‐associated pneumonia, further reinforcing the interplay between gut, lung, and brain (Cotoia et al. 2023). These findings raise the possibility that disruption to the lung‐brain axis may contribute to neurological impact such as cognitive impairment and neuroinflammation.

### 3.2 Emerging Axes: The Lung‐Gut‐Skin, Lung‐Gut‐Kidney, Lung‐Gut‐Liver, and Lung‐Gut‐Heart Axes

Unlike the gut‐lung and lung‐brain axes, direct evidence for lung crosstalk with the skin, liver, kidney, and heart remains comparatively preliminary. Across all four organ systems, however, an emerging body of evidence points to the gut as a common intermediary (Yang, Lin, et al. 2025; Galaris et al. 2022; Liu et al. 2023; Arab et al. 2025).

#### 3.2.1 Lung‐Gut‐Skin Axis

Lung‐gut‐skin crosstalk is thought to draw on the same broad mechanistic processes described above, with emerging evidence indicating that the gut microbiome facilitates a potential crosstalk between the lung and skin, affecting inflammation, immune responses, and disease outcomes (Yang, Lin, et al. 2025). As an example, Mendelian randomization studies further report associations between specific skin and gut taxa and COPD risk, with taxa including *Micrococcus* and *Kocuria* exhibiting protective effects against COPD, while *Corynbacterium* and *Acinetobacter* being identified as risk factors, though these findings reflect compositional associations with the disease status rather than direct evidence of microbial exchange between the skin and lungs (Luo et al. 2025). Nonetheless, such associations may remain critical to note, as skin microbiota are increasingly recognized to modulate immune programming beyond the localized skin site itself (Prescott et al. 2017) suggesting that microbial signals originating in the skin could potentially extend to distal organs, including the lungs, warranting further investigation. Studies to date also note our skin's association to microbial pathogens as a stimulant toward systemic inflammatory responses observed in the airways, including pulmonary Th17 responses (Deng et al. 2025; Deckers et al. 2018; Saunders et al. 2020). Furthermore, clinical observations from the intensive care unit (ICU) relating to severe pneumonia reveal a reduced skin bacterial diversity alongside increases in potential pathogens such as *Staphylococcus* and *Acinetobacter* on the skin. Many such pathogens were detected in the respiratory tract, highlighting the possibility of direct microbial exchange and their associated pathogenic risk (Lu et al. 2022). Beyond this, direct mechanistic links between skin and lung microbiota remain understudied, with recent reviews highlighting this as a largely unexplored area (Teaw et al. 2021). Overall, emerging data to date underscore a complex interaction between the lung and skin, where microbiomes have crucial roles in mediating immune signaling and barrier function. Such interplay ultimately affects susceptibility to lung disease and suggests potential fresh avenues for prevention and management in these associated disease states (Yang, Lin, et al. 2025).

#### 3.2.2 Lung‐Gut‐Liver Axis

The interaction mediated by microbiomes between the lung and liver is increasingly acknowledged in a number of disease settings, demonstrating the close integration between microbial perturbations, including diversity loss and shifts in taxa such as *Firmicutes* across the gut‐liver‐lung axis, and metabolic perturbations including oxidative stress‐driven alterations in microbial metabolites and disruption of both gut and alveolar‐capillary barrier integrity, and consequent hepatic inflammation and disrupted lipid metabolism, within and between organ systems (Galaris et al. 2022). Lung‐gut‐liver crosstalk appears to be bidirectional, often involving the systemic circulation of microbial products and inflammatory signals originating from one of the two organ systems (Herrero et al. 2020). Most of the evidence implies that lung‐liver crosstalk is gut mediated. For instance, in COVID‐19, multi‐omics profiling illustrates that alterations in gut microbiota (and their metabolites) identify liver dysfunction as a key extrapulmonary complication. Specific networks of microbes, metabolites, and cytokines are correlated with the severity of hepatic injury (Nagata et al. 2023). However, this association was observed in a cohort defined by respiratory infection, rather than direct measurement of a lung‐mediated pathway; thus, it is better characterized as evidence of gut‐liver linkage in the context of lung disease. Murine studies offer more direct support for a shared microbial axis and have shown that obesity induced by a high‐fat diet alters microbial populations in the gut, liver, and lungs. More specifically, this leads to lung microbiomes resembling those seen in the liver, expanding the *Firmicutes* phylum across all three organ sites and supporting a gut‐liver‐lung microbial axis (Galaris et al. 2022). Notably, *Staphylococcus*, *Streptococcus*, and *Finegoldia* were all consistently enriched across these organs, with *Staphylococcus* illustrating the strongest increase, raising the possibility that it serves as a pathogenic link between organs during metabolic stress. However, this compositional similarity across organs following a shared systemic exposure could denote parallel, independent responses to the same dietary trigger. Beyond this, a direct lung‐liver connection has also been experimentally demonstrated. Notably, exposure to environmental ozone introduces an additional dimension directly linking the lungs and the liver, as inhaled ozone compromises pulmonary barriers, allowing low inoculums of *Klebsiella pneumoniae* to colonize the lungs, enter the bloodstream, and migrate to the liver, where it may induce hepatic injury (Jia et al. 2025). This provides an experimentally demonstrated example of pulmonary barrier disruption translating into hepatic pathology. Taken together, this evidence suggests that the lung‐gut‐liver axis disruption may drive hepatic pathology, including impaired lipid metabolism and liver injury.

#### 3.2.3 Lung‐Gut‐Kidney Axis

The communication network linking the lungs and kidneys is undoubtedly influenced by the gut microbiome through the systemic circulation of microbial mediators. This newly emerging concept of a lung‐gut‐kidney axis, most commonly with the gut as intermediary, suggests that intestinal microbes affect pulmonary and renal health, establishing important connectivity between dysbiosis and systemic disease (Liu et al. 2023). In COPD, individuals without kidney dysfunction demonstrate a gut microbiota characterized by an increased *Firmicutes* and decreased *Bacteroidetes* (Li, Dai, et al. 2021). Conversely, individuals with COPD with concurrent kidney dysfunction demonstrate enrichment of *Bacteroidetes* (*Tannerella* and *Capnocytophaga*), *Fusobacteria*, and *Actinobacteria*, indicating that specific microbial change is associated with a combined lung‐kidney impairment (Saipova et al. 2025). Elevated systemic inflammation, including raised C‐reactive protein and TNF‐⍺, correlates with these microbial patterns and renal indices, reinforcing the association between gut dysbiosis, lung disease severity, and renal injury (Alobaidi 2025; van Iersel et al. 2025). These findings potentially indicate that gut microbial composition differs by comorbidity status in COPD, though they reflect a cross‐sectional association between gut dysbiosis, lung disease and renal injury, rather than an established lung‐kidney pathway. Complementary investigations including renal transplant recipients infected with coronavirus reveal a lung‐gut‐kidney interaction, where patients infected with novel coronavirus pneumonia (NCP) exhibit disrupted gut microbiomes, particularly an increased *Firmicutes* and *Actinobacteria* that in turn associate with elevated serum creatinine, poorer allograft function and altered gut‐lipid metabolites directly linking gut microbes to renal outcomes during respiratory infection (Wang et al. 2024). Beyond this gut‐mediated evidence, a more direct pathway has also been proposed. A clear example of direct microbial involvement is the viral invasion of renal podocytes documented in severe COVID‐19, with transmission electron microscopy revealing viral particles with characteristic spikes found within the podocytes of deceased COVID‐19 renal tissue suggesting that viral invasion may potentially contribute to capillary barrier dysfunction and proteinuria, both frequently observed in COVID‐19 (Abbate et al. 2020). The entry of SARS‐CoV‐2 into the systemic circulation paves way for the potential initiation of acute kidney injury (Pan et al. 2020). Given that viruses represent a recognized component of the broader microbiome, the virome (Cao et al. 2022), this finding may constitute a nascent example of direct microbial transit between the lung and kidney, and highlights viral‐mediated crosstalk as a promising, currently underexplored avenue for further investigation of this axis.

#### 3.2.4 Lung‐Gut‐Heart Axis

Growing evidence suggests human microbiome involvement in orchestrating functional and inflammatory crosstalk between the lungs and heart. Persistent alterations in microbial communities, especially within the gut, following COVID‐19, are associated with systemic immune responses, endothelial integrity, and cardiovascular markers, suggesting a plausible lung‐gut‐heart axis, though the lung's specific mechanistic contribution to this relationship has not been directly established (Arab et al. 2025). Evidence from systematic reviews broadens this concept to encompass a lung‐gut‐heart axis in long‐COVID (Arab et al. 2025) where pulmonary sequelae from this clinical state, including persistent inflammation and endothelial dysfunction, places strain on the cardiovascular system. Shared mechanisms, potentially exacerbated by gut dysbiosis, include heightened intestinal permeability allowing bacterial endotoxins to enter the bloodstream and perpetuate systemic inflammation which negatively impacts cardiopulmonary function. Increased levels of trimethylamine‐N‐oxide (TMAO) associate with endothelial dysfunction and thrombotic events, which in themselves represent mechanisms worsening cardiopulmonary damage (Haissman et al. 2017; Cimmino et al. 2025). Extensive expression of ACE2 across the gut, lungs, and heart further link viral entry, disruption of the renin‐angiotensin‐aldosterone system and inflammation mediated by the microbiome suggesting that targeting such axes may alleviate the long‐term cardiopulmonary consequences related to long‐COVID (De and Dutta 2022). Whether lung‐gut‐heart axis disruption is a cause or consequence of cardiovascular pathology remains elusive, but the convergence of gut permeability, TMAO signaling, and shared ACE2 expression contributes to outcomes such as endothelial dysfunction and thrombotic risk, though further studies would be required to ascertain this.

## 4 Extrapulmonary Disease as a Converging Outcome of Pulmonary‐Organ Axis Disruption

Chronic respiratory diseases carry a profound burden of comorbidity beyond the lung itself, with cardiovascular disease, renal disease, hepatic disease, and cognitive decline occurring at rates exceeding what age and shared risk factors such as smoking alone would predict, and this pattern is widely documented in COPD (Divo et al. 2012; Stratev and Fjeldstad 2023), though comorbidity burden is also recognized in asthma (Veenendaal et al. 2019) and interstitial lung disease (Jovanovic et al. 2022). Gut microbiome dysbiosis is a consistent feature of COPD specifically, with a reduced microbial diversity and modified taxonomic composition compared with healthy controls (Pan et al. 2025), and the axes described above provide a plausible mechanistic link through which this dysbiosis, whether originating from the gut or lung, may extend beyond the respiratory system to influence diseases in other organ systems.

Cardiovascular, renal and hepatic disease represent three additional extrapulmonary complications whose burden in chronic lung disease is increasingly well‐documented, complementing the mechanistic pathways described in the lung‐organ axes section. Cardiovascular disease is significantly more prevalent in COPD, with patients with severe‐to‐very‐severe airflow obstruction displaying more than double the odds of cardiovascular diseases relative to those with milder or no obstruction, independent of shared risk factors such as smoking (Mannino et al. 2008). Renal involvement follows a similar trend, whereby in the COSYCONET COPD cohort, chronic kidney disease was present in 7.1% of patients and was independently associated with more than twice the risk of mortality after adjusting for cardiovascular comorbidities, with declining renal function also independently associated with greater dyspnea severity and reduced capacity to exercise (Trudzinski et al. 2019). Hepatic involvement appears to be similarly frequent, whereby metabolic dysfunction associated with steatotic liver disease, which affects up to 25% of the general populace, is increasingly recognized as an underappreciated comorbidity in COPD, with one cohort reporting liver abnormalities consistent with this condition in 75% of patients (Al Dailaty et al. 2025; Viglino et al. 2017).

Cognitive decline represents a further extrapulmonary outcome with potential microbiome involvement, which extends the direct and gut‐mediated lung‐brain signaling mechanisms, reframing them from an acute neuroinflammatory event into a chronic process. Cognitive impairment is increasingly recognized as a comorbidity of COPD, conferring a higher risk of decline relative to those without COPD (Jing and Mao 2026). Repeated or sustained systemic inflammation and microbial translocation following chronic lung disease may eventually contribute to this impairment via mechanisms such as blood–brain‐barrier (BBB) disruption—a pathway directly demonstrated in long COVID, where sustained inflammation and elevated markers of BBB breakdown are associated with cognitive impairment (Greene et al. 2024).

## 5 Conclusions and Future Perspectives

Microbiome‐mediated pulmonary‐organ (including gut‐lung, lung‐brain, lung‐skin, lung‐liver, lung‐kidney, and lung‐heart) axes hold significant potential in transforming our understanding and management across several respiratory disease states. Such a fresh approach to evaluating pathogenesis, progression, and prognosis may offer novel directions for therapeutic targets and engagement. Progress in multi‐omics profiling, machine learning, and precision methodologies continues to enhance our understanding of these pulmonary‐organ axes (Wang, Yu, et al. 2025).

Therapeutic interventions in the gut including probiotics, prebiotics, and FMT have all shown the ability to restore microbial equilibrium, reduce inflammation, and improve outcomes in lung diseases such as asthma, COPD, and respiratory infections including pneumonia (Wang, Yu, et al. 2025; Sun et al. 2024). These approaches, while important, predominantly shape community‐level microbial composition non‐specifically. Beyond this, an increasing number of microbiome‐directed therapeutic strategies are now gaining traction.

Engineered live biotherapeutic products represent one such alternative, whereby synthetic biology approaches now permit commensal or probiotic bacteria to be genetically modified to detect localized disease signals, such as inflammatory mediators or hypoxia, and in response, produce and deliver therapeutic payloads directly at disease sites, functioning as living in situ drug‐delivery platforms (Srivastava and Lesser 2024). Although this technology remains mainly preclinical at this stage for pulmonary applications, its precision and reduced systemic exposure represent considerable progress from broad‐spectrum probiotic supplementation. Postbiotics offer a complementary, inert substitute, whereby inactivated microorganisms, cell fragments, or their metabolic by‐products preserve much of the immunomodulatory effects of live bacteria without added safety concerns (Salminen et al. 2021). Bacterial lysate preparation, for example, has been demonstrated to attenuate airway hyperresponsiveness and systemic inflammation in asthma, elucidating a stable, standardizable pathway to microbiome‐informed therapeutics (Węgrzyn et al. 2024).

Bacteriophage therapy, which selectively targets pathogenic bacteria while preserving commensals, is regaining clinical interest for respiratory infections amid rising antimicrobial resistance (Alshehri et al. 2026). Inhaled and nebulized phage preparations are progressively being used in trials for multidrug‐resistant lower respiratory tract infections, including for bronchiectasis, cystic fibrosis, and ventilator‐associated pneumonia; through there remains a paucity of large, randomized trials (Khosravi et al. 2024). Collectively, these approaches converge on precision microbiome modulation, tailoring interventions using multi‐omic and clinical profiles rather than applying one‐size‐fits‐all treatment protocols (Ratiner et al. 2024; Shahid 2025).

Apart from certain direct interactions between the lung and other organ systems including vagally‐mediated and microbial translocation‐based lung‐brain signaling (Liu et al. 2025; Ma et al. 2023; Villalba et al. 2023), direct microbial exchange between the lung and skin (Lu et al. 2022), and lung‐to‐liver bacterial translocation following pulmonary barrier disruption (Jia et al. 2025), the gut microbiome frequently acts as a central intermediate mediator where microbial metabolites and immune mediators derived from the gut are increasingly recognized for their key roles in influencing pulmonary inflammation, while concurrently impacting cardiovascular, renal, hepatic, and skin health through the systemic circulation of immune cells (Guo et al. 2023). Understanding such pulmonary‐organ axes allows us potential cross‐site interventions including targeted modulation of microbiota, dietary changes and/or therapies targeted at metabolites, opening up the potential to address comorbidities related to the respiratory system and the extrapulmonary disease burden associated with respiratory disease states as detailed above. Use of accessible distal‐organ microbiome activity, such as circulating metabolites, systemic inflammatory signatures, and multi‐omic profiling, to stratify patients with respiratory diseases presents new avenues for patient endo‐phenotyping beyond what “difficult to obtain” airway sampling can provide. This is particularly pertinent with strong emerging evidence linking gut microbiome alterations, which can be readily sampled through stool, with respiratory disease subtypes in asthma, COPD and idiopathic pulmonary fibrosis (Bowerman et al. 2020; Arrieta et al. 2015; Molyneaux et al. 2014). For organs such as the liver and brain, where direct microbiome sampling remains clinically impractical at present, future work should instead prioritize indirect, gut‐mediated connections between the lung and these organ systems, and the development of diagnostic and prognostic markers from circulating metabolites and multi‐omics data, rather than necessitating direct tissue access (Aragonès et al. 2020). Future work should emphasize the importance of understanding indirect gut‐mediated connections between the lung and other organ systems, while formulating integrative diagnostic and prognostic biomarkers utilizing multi‐omics technologies to better understanding with the aim of restoring respiratory and systemic health. While gut‐mediated pathways account for most of the pulmonary‐organ crosstalk described in this review, the handful of directly demonstrated lung‐organ connections identified warrant dedicated mechanistic studies to clarify their clinical contribution and significance.

Deciphering microbe‐mediated pulmonary‐organ crosstalk represents a paradigm shift in respiratory medicine and an emerging, exciting field of microbiology, as microbial metabolites and community dynamics are re‐positioned as central orchestrators, with a promise to better understand pathogenesis and develop integrated therapeutic approaches going beyond the lung and viewing lung diseases more holistically, including their diverse extrapulmonary complications.

## Funding

This research was supported by the National Research Foundation Singapore under its Open Fund‐Large Collaborative Grant (MOH‐001636) (S.H.C.) and administered by the Singapore Ministry of Health's National Medical Research Council; the Singapore Ministry of Health's National Medical Research Council under its Clinician‐Scientist Award (CSA) Investigator (INV) category (MOH‐001855) (S.H.C.) and Clinician‐Scientist Individual Research Grant (MOH‐001356) (S.H.C.) and the LKCMedicine Wong Peng Onn Postdoctoral Fellowship (#025152‐00001) (J.K.N.).

## Conflicts of Interest

S.H.C. has served on advisory boards for CSL Behring, Pneumagen Ltd., Zaccha Pte Ltd, Boehringer‐Ingelheim, GSK, Chiesi Farmaceutici and Sanofi, on DSMBs for Inovio Pharmaceuticals and Imam Abdulrahman Bin Faisal University and has received personal fees from Astra‐Zeneca, Boehringer‐Ingelheim, CSL Behring and Chiesi Farmaceutici, all unrelated to this work. All other authors have no potential conflicts of interest to disclose.

## Data Availability

Data sharing not applicable to this article as no datasets were generated or analysed during the current study.
